# Collective Intelligence Increases Diagnostic Accuracy in a General Practice Setting

**DOI:** 10.1177/0272989X241241001

**Published:** 2024-04-12

**Authors:** Matthew D. Blanchard, Stefan M. Herzog, Juliane E. Kämmer, Nikolas Zöller, Olga Kostopoulou, Ralf H. J. M. Kurvers

**Affiliations:** The University of Sydney, Sydney, Australia; Max Planck Institute for Human Development, Berlin, Germany; Department of Social and Communication Psychology, Institute for Psychology, University of Goettingen, Germany; Department of Emergency Medicine, Inselspital, Bern University Hospital, University of Bern, Switzerland; Max Planck Institute for Human Development, Berlin, Germany; Institute for Global Health Innovation, Imperial College London, UK; Max Planck Institute for Human Development, Berlin, Germany

**Keywords:** collective intelligence, decision support systems, diagnostic accuracy, general practice, medical diagnostics, wisdom of crowds

## Abstract

**Background:**

General practitioners (GPs) work in an ill-defined environment where diagnostic errors are prevalent. Previous research indicates that aggregating independent diagnoses can improve diagnostic accuracy in a range of settings. We examined whether aggregating independent diagnoses can also improve diagnostic accuracy for GP decision making. In addition, we investigated the potential benefit of such an approach in combination with a decision support system (DSS).

**Methods:**

We simulated virtual groups using data sets from 2 previously published studies. In study 1, 260 GPs independently diagnosed 9 patient cases in a vignette-based study. In study 2, 30 GPs independently diagnosed 12 patient actors in a patient-facing study. In both data sets, GPs provided diagnoses in a control condition and/or DSS condition(s). Each GP’s diagnosis, confidence rating, and years of experience were entered into a computer simulation. Virtual groups of varying sizes (range: 3–9) were created, and different collective intelligence rules (plurality, confidence, and seniority) were applied to determine each group’s final diagnosis. Diagnostic accuracy was used as the performance measure.

**Results:**

Aggregating independent diagnoses by weighing them equally (i.e., the plurality rule) substantially outperformed average individual accuracy, and this effect increased with increasing group size. Selecting diagnoses based on confidence only led to marginal improvements, while selecting based on seniority reduced accuracy. Combining the plurality rule with a DSS further boosted performance.

**Discussion:**

Combining independent diagnoses may substantially improve a GP’s diagnostic accuracy and subsequent patient outcomes. This approach did, however, not improve accuracy in all patient cases. Therefore, future work should focus on uncovering the conditions under which collective intelligence is most beneficial in general practice.

**Highlights:**

- We examined whether aggregating independent diagnoses of GPs can improve diagnostic accuracy.
- Using data sets of 2 previously published studies, we composed virtual groups of GPs and combined their independent diagnoses using 3 collective intelligence rules (plurality, confidence, and seniority).
- Aggregating independent diagnoses by weighing them equally substantially outperformed average individual GP accuracy, and this effect increased with increasing group size.
- Combining independent diagnoses may substantially improve GP’s diagnostic accuracy and subsequent patient outcomes.

Diagnostic errors are prevalent in clinician practice. It is estimated that 5.2% of hospital mortality in the United Kingdom results from preventable medical errors, such as incorrect diagnoses.^1^ Diagnostic errors may also contribute to management errors, such as incorrect prescriptions, which can cause preventable harm to patients.^2–4^ General practitioners (GPs) are typically the first point of contact between patients and specialists. They perform an important role in the early detection of debilitating and life-threatening diseases. Thus, decreasing diagnostic errors in a general practice setting is crucial for improving patient outcomes.

Considerable effort has been invested in the development of competence-boosting interventions that aim to reduce GP errors, such as computerized decision aids,^5–8^ checklists,^9–11^ and electronic records.^12–14^ These approaches aim to increase the diagnostic accuracy of individual decision makers. An alternative, and potentially complementary, approach for boosting diagnostic accuracy—that we will investigate here—is to harness the wisdom of multiple decision makers.

Collective intelligence broadly refers to the finding that multiple minds generally produce better outcomes than individual minds do, as shown in a wide range of domains (e.g., Hill,^15^ Laughlin,^16^ and Woolley et al.^17^). These outcomes can be produced via various methods, such as interacting consensus-seeking groups^18–23^ or the pooling of multiple independent judgments. The latter is known as the *wisdom-of-crowds effect*,^24–27^ which describes the observation that aggregating independent judgments generally outperforms the average individual group member and in some cases even the best member.^20,28–30^

The pooling of independent decisions has been successfully applied to a diverse range of tasks, including the prediction of election outcomes,^31^ memory retrieval,^32^ fingerprint analysis,^33^ false news identification,^34^ and medical decision making.^35^ Within medicine, it has mostly been applied to well-defined environments (i.e., low time pressure and complete information) such as interpreting mammograms,^36^ detecting skin lesions,^37^ identifying lower back pain,^38^ and predicting the likelihood of a future positive bone scan.^39^ However, there is a paucity of research that has applied this approach to more ill-defined medical environments,^35,40,41^ such as emergency medicine (i.e., high time pressure and incomplete information^42^) or general practice. GPs routinely face a diverse range of symptoms and illnesses and operate in an environment with high uncertainty, incomplete information, and variable time pressure.^43^ Typically, patients seek a diagnostic decision from a single GP but may also have the opportunity to seek independent diagnoses from multiple GPs. Here, we investigated the effectiveness of pooling independent diagnoses from multiple GPs for improving diagnostic accuracy and identified the conditions associated with the greatest accuracy improvement. This is relevant both from the perspective of GPs aggregating decisions as well as patients receiving different recommendations from different GPs. We return to these perspectives in the discussion.

In addition, we compared the benefit of pooling independent decisions with the benefit of using a decision support system (DSS). The DSS we investigated was designed by Kostopoulou et al. as part of the EU FP7-funded TRANSFoRm project. Following a series of studies,^6,7,44,45^ the DSS in its final form provides diagnostic suggestions to GPs early on in the consultation, namely, as soon as they enter a reason for the encounter. The list of suggestions is updated as GPs enter further information that they collect during the consultation. In general, differential diagnosis generators have been shown to increase the diagnostic accuracy of GPs by 6 to 9 percentage points,^5–8^ by increasing the number of diagnostic hypotheses under examination, encouraging a broader information search and reducing premature closure.^46–48^ Finally, we investigated the potential benefits of combining the 2 approaches by pooling independent decisions that are made with the assistance of a DSS. To our knowledge, no previous research has yet examined the combined influence of these 2 approaches on decision accuracy.

Our main aim was to examine whether pooling independent decisions could increase diagnostic accuracy in a general practice setting. Using 2 previously published data sets, we investigated the performance of different collective intelligence rules (plurality, confidence, and seniority), which were used to aggregate the independent diagnoses of multiple GPs. Based on previous research in an emergency medicine setting,^42^ we expected that all 3 collective intelligence rules would improve diagnostic accuracy as compared with average individual accuracy. Moreover, we expected that the plurality rule would outperform the confidence and seniority rule, especially at higher group sizes (see also Kämmer et al.^42^). We had no a priori expectation as to whether pooling independent decisions that are made with the assistance of a DSS would outperform pooling unassisted independent decisions (or individual decisions assisted by a DSS). Alongside our comparison of the overall performance of the different aggregation rules, we also studied which individual patient cases profited more (or less) from aggregation. Parallel to findings in binary decision making,^49–52^ we expected that aggregation would work well for patient cases in which the most common diagnosis given is the correct one and cases in which GPs made different errors (i.e., uncorrelated votes).

## Method

Our analyses were conducted on data from 2 previously published studies. In study 1 (hereafter called the *vignette data set*), 260 GPs independently diagnosed patient cases in a vignette-based study.^6^ In study 2 (hereafter called the *actor–patient data set*), 30 GPs independently diagnosed patient actors in a patient-facing study.^7^ In both data sets, GPs diagnosed fictitious patients with or without the aid of a DSS.

### Experimental Procedures

The task for GPs in both studies was to diagnose fictitious patients with 1 of 3 presenting problems: chest pain, abdominal pain, or dyspnea. Each case had a unique correct diagnosis. The vignette data set consisted of a control condition and 2 DSS conditions (early and late DSS). The actor–patient data set consisted of a control condition and 1 DSS condition (early DSS). In the control condition, GPs diagnosed patients without the DSS. In the DSS condition of the vignette study, the DSS provided diagnostic suggestions either early or late in the consultation before GPs entered their final diagnosis for a case. Below, we describe both studies in more detail. For full details, we refer to the original publications.

#### Vignette data set (*n* = 260)^6^

The vignette study employed a between-subject design with 3 conditions: 1) control, 2) early DSS, and 3) late DSS. The experimental task was administered online and comprised 9 vignette patient cases, presented in a random order to all participants in each condition. Such vignettes are considered a valid tool for measuring the quality of clinical practice.^53^ Participants received training on 1 practice case before proceeding to the 9 test cases. For each case, participants in the control condition were presented with information about a simulated patient including the reason for their encounter with a GP. They could request additional information about the patient’s history, physical examinations, and investigations, which was—upon request—displayed on their screen. When participants wanted to end the consultation, they entered their diagnosis as free text, their level of confidence (range: 1–8), and selected a management decision from a predefined list (refer, prescribe, arrange follow-up, give advice, or wait and see). Participants were then asked to specify their management decision (e.g., if they chose to prescribe medication, they also entered the type of medication). They were then presented with the next patient case.

In the early DSS condition, after reading the patient vignette, participants were additionally presented—for a minimum of 20 s—with a list of diagnostic suggestions appropriate for the patient’s age, sex, and presenting problem. When they confirmed they had read the list of possible diagnoses, it disappeared and they could begin requesting additional information about the patient. In the late DSS condition, the list of diagnostic suggestions was presented after participants had submitted a preliminary diagnosis and management decision. After seeing the list, participants could request further information about the patient and change their diagnosis and/or management decision.

#### Actor–patient data set (*n* = 30)^7^

The actor–patient study used a within-subject design with 2 conditions: 1) control and 2) early DSS. A different set of 6 patient cases was assigned to each condition. The experiment took place at King’s College London in a room set up to resemble a GP’s consultation room in the United Kingdom. The fictitious patient cases were presented by actors trained in medical communication. Similar to real patient consultations, participants discussed the presenting problem with the patient, gathered additional information (e.g., medical history and other symptoms), and ordered further investigations. If they requested investigations that did not require specialist referral then the results were received at the end of the consultation before entering a diagnosis. Participants could not perform physical examinations on patients, but they were able to indicate which examinations they would perform, and the fictitious patient provided the results immediately. Once the consultation ended, participants entered their diagnosis, confidence level (range: 1–10), and selected and specified a management decision (refer, prescribe, arrange follow-up, give advice, or wait and see).

In the early DDS condition, before starting the task, participants received 20 to 40 min of training to use the DSS. After participants entered the patient’s presenting problem, they were shown a list of diagnostic suggestions relevant for the patient’s age, sex, and presenting problem. When participants acknowledged they had read the list of suggestions, it disappeared but they could recall the list anytime by pressing a button. Participants were encouraged to code additional symptoms obtained from the patient, which updated the list of suggestions provided by the DSS.

### Participants

#### Vignette data set

Participants were 297 GPs (46% female, 54% male; mean years of GP experience: 8.8) recruited in the United Kingdom. Across the 3 conditions, 33 GPs were missing 1 or more confidence rating(s) due to a technical error (control = 12, early DSS = 10, late DSS = 11), and 4 GPs were missing information concerning their years of experience working as a GP (early DSS = 2, late DSS = 2). Given that we could not simulate the confidence or seniority rules for these GPs, they were excluded from all analyses. The final sample thus contained 260 GPs (46% female, 54% male; mean years of GP experience: 9.3) across the 3 conditions (control = 87, early DSS = 87, late DSS = 86). See Table 1 for more demographic details.

**Table 1:** Characteristics of General Practitioners in Each Data Set

Characteristic	Vignette	Actor–Patient
Total	260	30
Gender		
Male	140	15
Female	120	15
Years of experience		
≤10	168	17
11–20	47	5
≥21	45	8
Conditions		
Control	87	30
Early DSS	87	30
Late DSS	86	—

DSS, decision support system.

#### Actor–patient data set

Participants were 34 GPs (50% female, 50% male; mean years of GP experience: 12.7) recruited in the United Kingdom. GPs completed half of the cases in a control condition (i.e., no DSS) and the other half in an early DSS condition (counterbalanced across participants). Four GPs received a different counterbalancing procedure, making it difficult to simulate groups for these 4 GPs, so they were excluded from the analyses. Our final sample thus contained 30 GPs (50% female, 50% male; mean years of GP experience: 12.3). See Table 1 for more demographic information.

### Standardizing Diagnoses

In both data sets, GPs entered their diagnoses using free text so their responses could differ in various ways. This variance constituted differences in discrete diseases but also differences in spelling, capitalization, and synonyms for the same disease. Before conducting the computer simulations, these differences were removed so unique diagnoses referred to unique diseases. The research team, which included an experienced GP, standardized the diagnoses by grouping synonyms of the same disease together so we could use a single standardized term to describe each group of diagnoses. The collective intelligence rules were then applied to these standardized diagnoses.

### Accuracy Criterion

Diagnostic accuracy was the accuracy measure for both data sets. This binary measure indicated whether a diagnosis made by a GP for a particular case was correct or incorrect. All scenarios contained at least 1 piece of evidence (e.g., an examination result or a diagnostic test result) that was strongly predictive (or confirmatory) of only 1 of the competing diagnoses. Only 1 diagnosis was consistent with all the available information in each scenario. Note that participants in the study would usually request a subset of the available information and not necessarily the most diagnostic piece of evidence.

To quantify the relationship between confidence/seniority and accuracy, we used Bayesian mixed-level logistic regression models using the brm() function from the *brms* R package (version 2.20.4) using its default priors (and R version 4.3.2). We fitted accuracy (incorrect v. correct) as a binomial response variable and confidence, seniority, and condition (i.e., control, early DSS, late DSS) and the interaction between confidence:condition and seniority:condition as population-level (“fixed”) effects. GP identity and case identity were included as group-level (“random”) intercepts. We ran a separate model for the vignette and the actor–patient data set. For each model, we ran 3 chains in parallel with 6,000 iterations, of which the first 3,000 were discarded as burn-in. Visual inspection of the Markov chains and the Gelman–Rubin statistic $\left(\hat{R}\right)$ indicated that all Markov chains converged. As inference criterion, we evaluated whether the effects were credibly different from 0 (either the main effects or their interaction). See Supplementary Tables S1 and S2 for the full regression results.

### Simulating Virtual Groups

For each combination of 1) data set, 2) condition, and 3) group size, we created all possible unique virtual groups (i.e., groups with different group members)—unless the number of unique groups for a given combination was greater than 6,000 (in those cases, we randomly sampled 6,000 unique groups to reduce calculation time). For the simulations, we used R (version 4.3.2).

### Collective Intelligence Rules

For each virtual group, we selected 1 response for each case by applying the following collective intelligence rules:

1. The plurality rule selected the most common diagnosis chosen by the group members.^42,54^ This rule performs well when the correct diagnosis is the most chosen diagnosis. In case of a tie (e.g., two diagnoses with equal amount of support), we randomly sampled one diagnosis from these ties. Any ties in the next two rules were also solved by random sampling.
2. The confidence rule selected the diagnosis chosen by the group member with the highest confidence level.^19,29,42,55^ This rule generally performs well when confidence is positively correlated with accuracy. This rule serves as a benchmark to illustrate what is achievable when betting solely on the most confident diagnosis. In addition, we implemented a 3-person confidence rule, aggregating the diagnoses of the 3 most confident group members using a plurality rule.
3. The seniority rule selected the diagnosis chosen by the most experienced group member.^42^ We used years of experience as a proxy for expertise.^56^ This rule performs well when seniority is a good proxy for accuracy (i.e., they are positively correlated). In addition, we implemented a 3-person seniority rule, aggregating the diagnoses of the 3 most senior group members.

## Results

**Figure 1:**
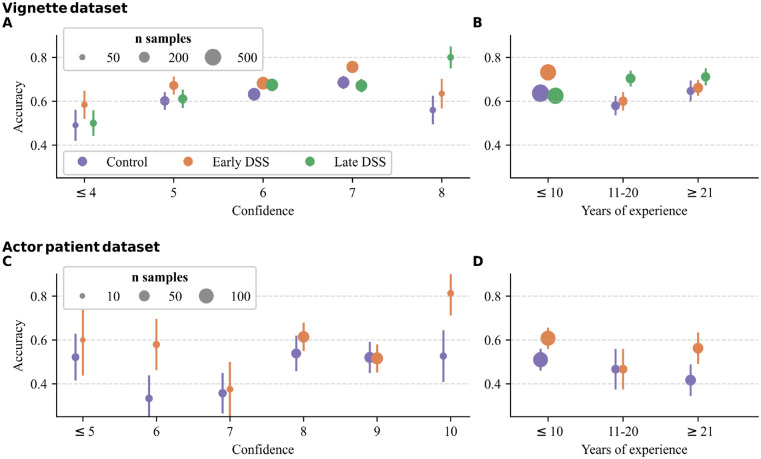
The relationship between diagnostic accuracy and confidence rating (A and C) and years of general practitioner (GP) experience (B and D) for the vignette and actor–patient data sets. Dots and error bars show the mean and standard error of the mean. The size of the dots corresponds to the number of observations. Note that different scales were used for confidence ratings: in the vignette data set, confidence ratings ranged from 1 to 8, and in the actor–patient data set, they ranged from 1 to 10.

Before applying the collective intelligence rules, we examined the distributions of confidence and seniority and how they were related to diagnostic accuracy in each data set. In both data sets, low confidence ratings were infrequently used, and most GPs reported 10 or fewer years of experience in general practice (Figure 1; Supplementary Figure S1).

In the vignette data set, higher confidence values were associated with higher accuracy levels (*β* [CI] = 0.21 [0.05, 0.38]; Figure 1A), and there was a weak, but not reliably negative, effect of seniority on accuracy (*β* [CI] = −0.01 [−0.04, 0.01]; Figure 1B). The interaction terms were not reliably different from zero (see Supplementary Table S1 for full regression results).

In the actor–patient data set, there was no association between confidence and accuracy (*β* [CI] = −0.01 [−0.24, 0.21]; Figure 1C) and a weak, but not reliably negative, effect of seniority on accuracy (*β* [CI] = −0.03 [−0.07, 0.01]; Figure 1D). The interaction terms were not reliably different from zero (see Supplementary Table S2 for full regression results).

Taken together, these patterns suggest that selecting diagnoses based on confidence may have a (weak) positive effect on diagnostic accuracy, while selecting diagnoses based on seniority may have little (or even a negative) effect on diagnostic accuracy.

**Figure 2:**
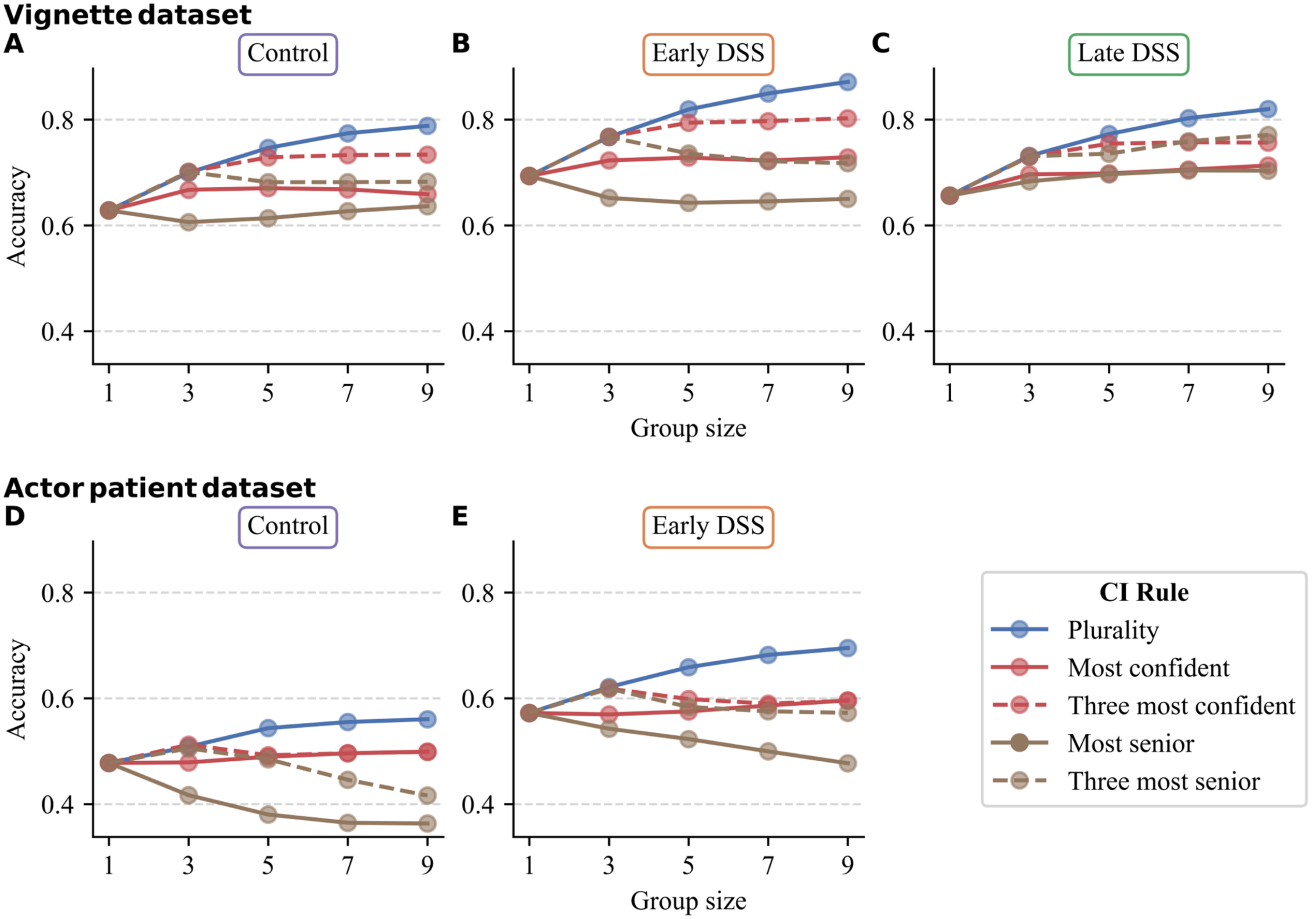
Mean diagnostic accuracy for each of the 5 collective intelligence rules per group size for the (A, D) control, (B, E), early decision support system (DSS), and (C) late DSS condition in both data sets. Group size 1 represents the average individual accuracy per condition.

Figure 2 shows the results of applying the collective intelligence rules. Across all 5 conditions, the plurality rule consistently outperformed average individual accuracy, and this benefit increased with group size. In both data sets, diagnostic accuracy was highest when the plurality rule was combined with the early DSS. The 3-most-confident rule also increased performance in the vignette data set compared with individual accuracy but was slightly worse than the plurality rule. In the actor–patient data set, both confidence rules did not lead to improvements. The seniority rules generally decreased performance, especially at larger group sizes in the actor–patient data set. In both data sets, the lowest performance was achieved when the seniority rule was used in the control condition. In summary, the plurality rule consistently outperformed 1) single GPs, 2) the confidence rules, and 3) the seniority rules.

**Figure 3:**
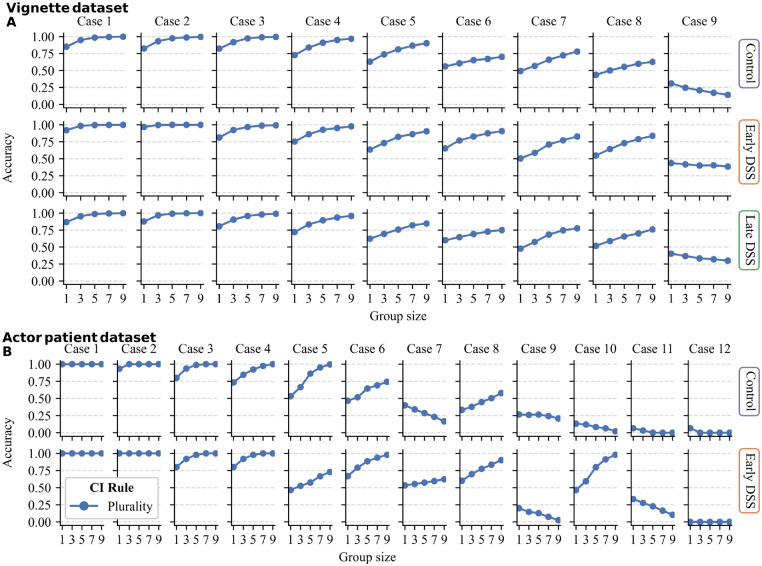
Performance of the plurality rule for each case and in each condition for the (A) the vignette dataset and (B) the actor-patient dataset. Within each data set, cases are arranged (from left to right) based on the mean individual accuracy in the control condition, with the highest (lowest) mean individual accuracy on the left (right).

Next, we investigated the performance at the case level, focusing on the plurality rule because only this rule consistently outperformed average individual accuracy. Figure 3 shows the performance of the plurality rule for each case and condition. In the vignette data set, across all 3 conditions, the plurality rule increased diagnostic accuracy with increasing group size in 8 of 9 vignettes and decreased performance in only 1 vignette. In the actor–patient data set, the results were more mixed. Here, the plurality rule decreased performance in the control condition in 5 of 12 cases and in the early DSS condition in 2 of 12 cases. In the discussion we further discuss these results and examine the conditions under which we expect the plurality rule to either promote or reduce accuracy in the context of GP decision making.

Supplementary Figures S2 and S3 show the performance of the confidence and seniority rules across cases. For both we do not see an obvious relationship between their performance and case difficulty.

**Figure 4:**
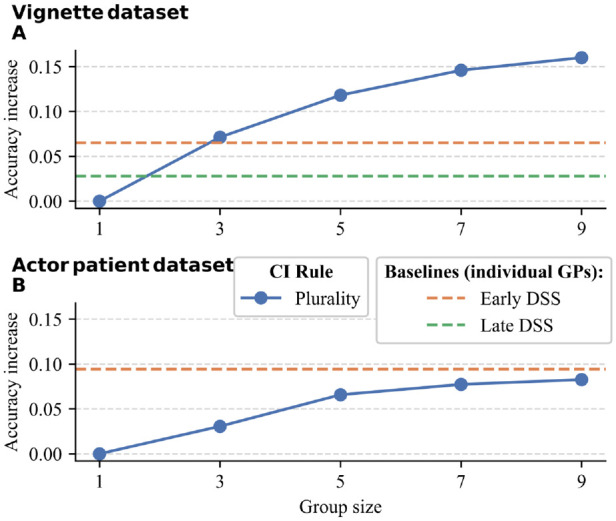
The increase in diagnostic accuracy (as compared with average individual accuracy) for the plurality rule (blue line) in (A) the vignette dataset and (B) the actor-patient dataset. The baselines for the early decision support system (DSS; orange line) and the late DSS (green line) correspond to the average accuracy increase for individual GPs in that condition.

Next, we compared the benefits of pooling decisions to the benefits of the DSS, focusing again on the plurality rule. Figure 4 shows the absolute increase in diagnostic accuracy (as compared with average individual accuracy in the control condition) for the plurality rule and for single GPs in the DSS conditions. At a group size of 3 in the vignette data set, the plurality rule performed similarly to single GPs with an early DSS. At larger group sizes, the plurality rule led to consistently higher performance than the early (or late) DSS. In the actor–patient data set; however, the early DSS led to consistently higher performance than the plurality rule. At the highest group size, the performance of the plurality rule approached that of individuals’ performance having access to the early DSS.

**Figure 5:**
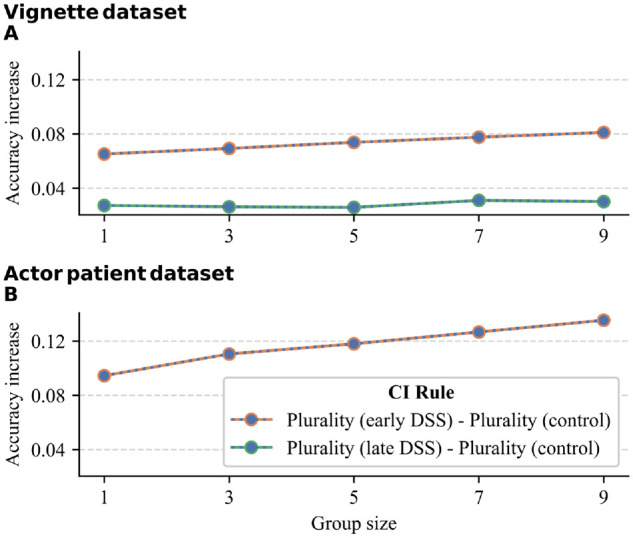
The interaction effect of collective intelligence, decision support system (DSS), and group size on diagnostic accuracy in (A) the vignette dataset and (B) the actor-patient dataset.

Lastly, we investigated whether the benefit of pooling decisions would be more pronounced when group members used a DSS compared with when group members did not. For each data set, group size, and DSS condition, we computed the difference in accuracy between the plurality rule in the DSS condition and the accuracy of the plurality rule in the respective study’s control condition. Figure 5 shows the results. In all comparisons, combining the plurality rule with the DSS condition rendered higher accuracy than the plurality rule by itself (i.e., there was synergy between both). In addition, in the early DSS condition in both data sets, increasing group size strengthened this effect, suggesting the benefit of collective intelligence was more pronounced with increasing group size when combined with an early DSS. For the late DSS condition in the vignette data set, this effect was absent.

## Discussion

Our results revealed that the plurality rule consistently outperformed the average individual, the confidence rules, and the seniority rules, and the benefit of this approach increased with group size. In fact, there was no condition in which the confidence or seniority rules performed better than the plurality rule in any of the settings (2 control and 3 DSS conditions across 2 data sets). The confidence rules also tended to increase diagnostic accuracy above the individual level; however, this benefit was smaller and less consistent than the plurality rule’s increase in accuracy. Confidence was, indeed, not well aligned with accuracy (see Figure 1). For the confidence rule to be effective, it is essential that confidence is positively correlated with accuracy and that participants provide confidence ratings on a common scale.^27^ In our study, as with many other medical studies, the use of Likert scales to measure confidence may have been problematic. These scales are notorious for being interpreted differently between raters (e.g., a confidence rating of “5” may have a very different meaning to different raters). One way to reduce this issue is to elicit subjective probabilities.^57^ Another persistent problem in the medical domain is overconfidence.^58^ Although we were not able to directly test for overconfidence due to the Likert scale, the abundance of relatively high confidence ratings—especially in light of individual accuracy—may hint at the possibility that overconfidence could have played a role in the data sets we analyzed. Taken together, this shows the challenge of using metacognitive data, such as confidence, for collective intelligence approaches in the medical domain.

Contrary to our expectation, and to a previous study,^42^ the seniority rule performed worse than individuals in both data sets because GPs with more years of experience were—if anything—less accurate than GPs with fewer years of experience (Figure 1). This relationship may have occurred because more experienced clinicians have a larger pool of similar patient cases available in memory,^59^ which may bias the process of generating diagnostic hypotheses,^6^ or it may be the result of more experienced physicians being further out from their medical training.

The plurality rule showed more potential than the confidence and seniority rules, as it outperformed those rules in all conditions. However, we did find substantial variation in the performance of the plurality rule between individual cases and conditions (Figure 3). How can this variation be understood? In binary decision making, the majority rule typically increases (decreases) accuracy whenever the average individual accuracy is above (below) 50%.^49–51^ In a similar way, we observed that the performance of the plurality rule generally decreased when case difficulty increased (defined as the average individual accuracy of a case). Moving from left to right in Figure 3 shows increasingly harder cases and increasingly poorer performance of the plurality rule. This can also explain the differences between data sets and conditions in the plurality rule performance. The average individual accuracy was substantially higher in the vignette study (control: 63%; early DSS: 69%) than the actor–patient data set (48% and 57%, respectively). The higher individual accuracy in the vignette study can explain why the plurality rule led to a higher overall increase in performance in this data set than the actor–patient data set (see Figure 2). Likewise, within the actor–patient data set, the plurality rule worked better in the early DSS condition than in the control condition, most likely due to the higher individual accuracy in the former.

Individual accuracy is, however, not the only factor determining the performance of the majority rule. The other key factor is the correlation of errors.^49,50^ Intuitively, when individuals make different errors, it is more likely that these are averaged out at the collective level, but if individuals make the same error (i.e., many support the same incorrect diagnosis), this is less likely. In the supplement, we show how the combination of individual accuracy and error correlation drive performance across cases. In a nutshell, the plurality rule performs well when the correct diagnosis is the most suggested diagnosis by individual diagnosticians out of all the suggested diagnoses. And, in these situations, the plurality rule works even better when the GPs make different errors, rather than the same ones (see also Supplementary Figure S4).

Our results revealed that the benefit of collective intelligence can exceed that of a DSS, but this outcome is not guaranteed. This was the case for the vignette data set but not the actor–patient data set. In the actor–patient data set, the benefit of collective intelligence approached, but did not reach, that of a DSS. Combining collective intelligence with a DSS produced the highest accuracy in both studies (i.e., we found an interaction between using an early—but not a late—DSS and the group size of the plurality rule). This interaction effect was stronger in the actor–patient data set. Our encouraging results for combining individual decisions made with the assistance of a DSS shows that both approaches could be synergistic and merit further research.

There are 2 important costs attributable to the implementation of a collective intelligence approach: 1) time and 2) financial resources. Referring a patient to multiple GPs requires a substantial amount of additional time to determine a diagnosis. Depending on the patient’s presenting symptoms, this additional time may exacerbate a patient’s physical and/or psychological suffering or may pose a risk to the efficacy of treatment. Our results indicate that the benefit of collective intelligence is similar when a patient’s case is presented as a vignette or face to face. Therefore, the initial GP could disseminate a description of a patient’s case to 2 (or more) other GPs for their opinion. An important question remains, who is best suited to aggregate multiple diagnoses: the initial GP, an additional independent GP, or the patient? A recent study investigated how single diagnosticians take up advice when receiving the collective-intelligence output from a group of previous raters in an open-ended medical-diagnostics task and found that single diagnosticians had higher diagnostic accuracy when receiving such advice.^60^ Second, a collective intelligence approach also incurs greater financial costs and places a greater burden on medical resources. We were not able to evaluate these costs in the present study. These are important next steps in evaluating the viability of applying collective intelligence to GP decision making and broadly to other medical domains.

Our findings come with several other limitations. First, they are based on data collected in a simulated, experimental setting not a real general practice environment. While the 2 experiments^6,7^ captured important characteristics of general practice, the real-world setting is more complex, with a broader range of possible diseases and greater uncertainty. Future research should apply collective intelligence to real-world data sets. When evaluating medical diagnoses it is important to distinguish between diagnostic errors (process) and the harm resulting from those errors (i.e., outcomes; Newman–Toker and Pronovost^4^). The consequences of diagnostic errors do not always lead to negative outcomes of equal severity. For example, misdiagnosing a pulmonary embolism as pneumonia would lead to antibiotic treatment and likely fluid restriction, both unnecessary or even detrimental for patients with a pulmonary embolism. In contrast, misdiagnosing a pulmonary embolism as a myocardial infarction would, despite the incorrect diagnosis, still imply thrombolytic therapy together with the application of oxygen and monitoring and/or pharmaceutically supporting cardiac output. We were not able to evaluate the outcomes of diagnostic errors in this study. Lastly, there is always a level of subjectivity involved with standardizing diagnoses, which may have affected our results, as the accuracy of the collective intelligence rules depended on the distribution of responses. A recently developed method automatically links free-text diagnoses to known entries in a medical ontology (i.e., SNOMED Clinical Terms; Kurvers et al.^41^). Using such approaches would help to reduce the level of subjectivity in future studies.

In conclusion, our results suggest that a carefully selected collective intelligence approach may increase diagnostic accuracy in a general practice setting, especially when combined with a DSS. In doing so, this approach may substantially reduce preventable diagnostic errors and litigation that may arise from those errors and improve patient outcomes in a GP setting.

## Supplemental Material

sj-docx-1-mdm-10.1177_0272989X241241001 – Supplemental material for Collective Intelligence Increases Diagnostic Accuracy in a General Practice Setting Supplemental material, sj-docx-1-mdm-10.1177_0272989X241241001 for Collective Intelligence Increases Diagnostic Accuracy in a General Practice Setting by Matthew D. Blanchard, Stefan M. Herzog, Juliane E. Kämmer, Nikolas Zöller, Olga Kostopoulou and Ralf H. J. M. Kurvers in Medical Decision Making
