# Supplementary material for: Collective Intelligence Increases Diagnostic Accuracy in a General Practice Setting
Source: Med Decis Making. 2024 Apr 12;44(4):451–62. doi: 10.1177/0272989X241241001 (PMC11102639; doi:10.1177/0272989X241241001)
Supplement: sj-docx-1-mdm-10.1177_0272989X241241001 – Supplemental material for Collective Intelligence Increases Diagnostic Accuracy in a General Practice Setting [file sj-docx-1-mdm-10.1177_0272989X241241001.docx]

**Supplementary Materials**

Belonging to

**Collective Intelligence Increases Diagnostic Accuracy in a General Practice Setting**

Matthew D. Blanchard, Stefan M. Herzog, Juliane E. Kämmer, Nikolas Zöller, Olga Kostopoulou, and Ralf H. J. M. Kurvers

**The conditions determining whether the plurality rule improves/reduces accuracy**

The key factor governing whether the plurality rule leads to an increase or decrease in accuracy is whether the correct diagnosis is the must-supported diagnosis among all diagnoses for a given case or not. Whenever the correct diagnosis is the most-supported diagnosis for a given case, increasing group size will increase diagnostic accuracy. Vice versa, whenever the most-supported diagnosis is an incorrect diagnosis, increasing group size will decrease diagnostic accuracy. In other words, the plurality rule amplifies the diagnosis supported by most GPs. Fig. S2 shows how often each diagnosis was given per i) case, ii) condition, and iii) dataset. The correct diagnosis is indicated with a pink bar. To illustrate the above, take case 7 in the actor-patient dataset. In the control condition, an incorrect diagnosis (ibs) is the most-supported diagnosis, hence the plurality rule reduces performance for this case in this condition (as indicated by a reduction in accuracy with group size for case 7 in the control condition in Fig. 3B). However, in the early Decision Support System (DSS) condition, the correct diagnosis (ovarian cancer) is the most-supported diagnosis (Fig. S2). This explains why in the DSS condition, the plurality rule leads to an increase in accuracy for this case (Fig. 3B).

How quickly the plurality rule leads to an increase (or decrease) in performance depends on the distribution of errors (i.e., incorrect diagnoses). Generally speaking, if the most-supported diagnosis is the correct one (and we thus expect the plurality rule to increase performance), the strength of increase in accuracy with group size will increase the more the incorrect responses are distributed over more (incorrect) diagnoses. To illustrate, we again turn to Case 7 in the early DSS condition in the actor dataset. As explained above, the correct diagnosis is the most-supported diagnosis, hence, we observe an increase in accuracy with group size (Fig. 3B). However, this increase is relatively weak, because there is one incorrect diagnosis (ibs) which also received substantial support. Hence, the plurality rule will still regularly arrive at this incorrect diagnosis. Now take Case 10 in this condition; here the correct diagnosis (tb) was also the most-supported diagnosis, explaining the increase in accuracy with group size for this case (Fig. 3B). However, the increase in accuracy is relatively strong. This is because there are many incorrect diagnoses given but all with low support. The plurality rule will thus often converge on the correct diagnosis because all incorrect diagnoses only have low (individual) support.

**Supplementary Figures**


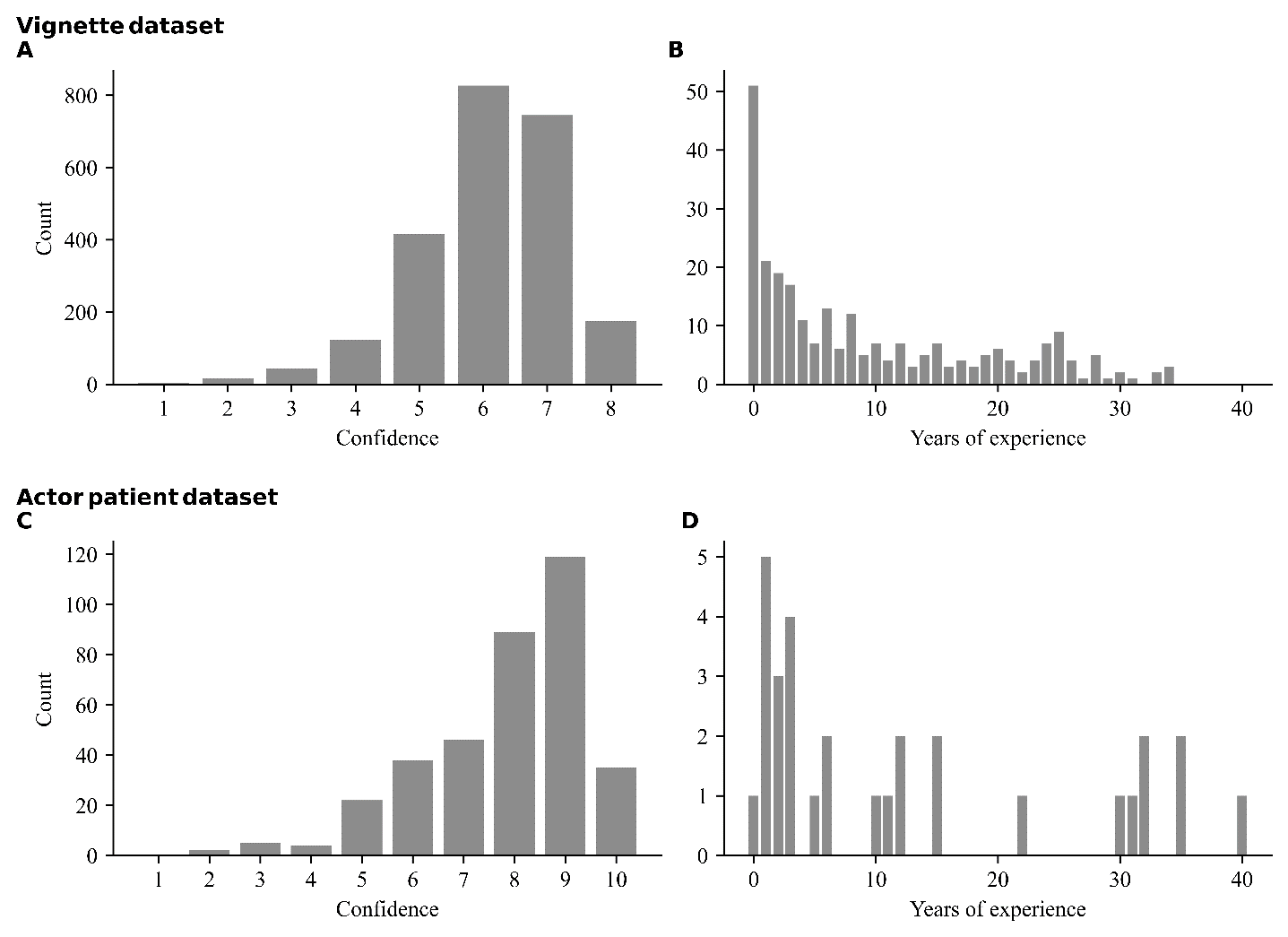


**Supplementary Figure 1**: The distribution of (A,C) confidence ratings and (B,D) GPs' years of experience for both datasets.


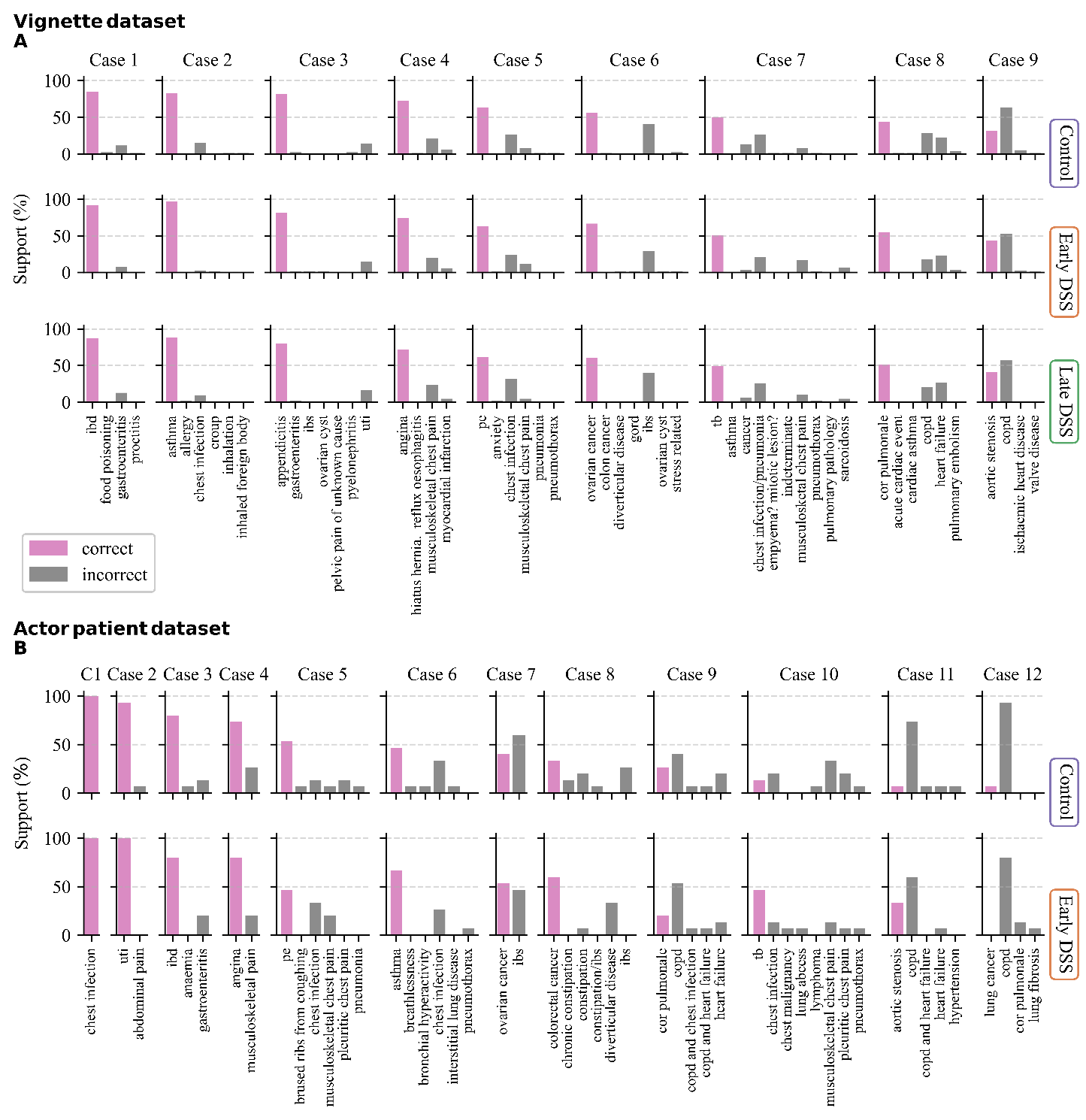


**Supplementary Figure 2**: The y-axis shows the percentage of GPs who provided a given diagnosis (x-axis) per case, condition, and dataset. Within each dataset, cases are arranged (from left to right) based on the mean individual accuracy in the control condition, with the highest (lowest) mean individual accuracy on the left (right). This order is identical to Main Figure 3. The pink-colored bar shows the correct diagnosis of a case.


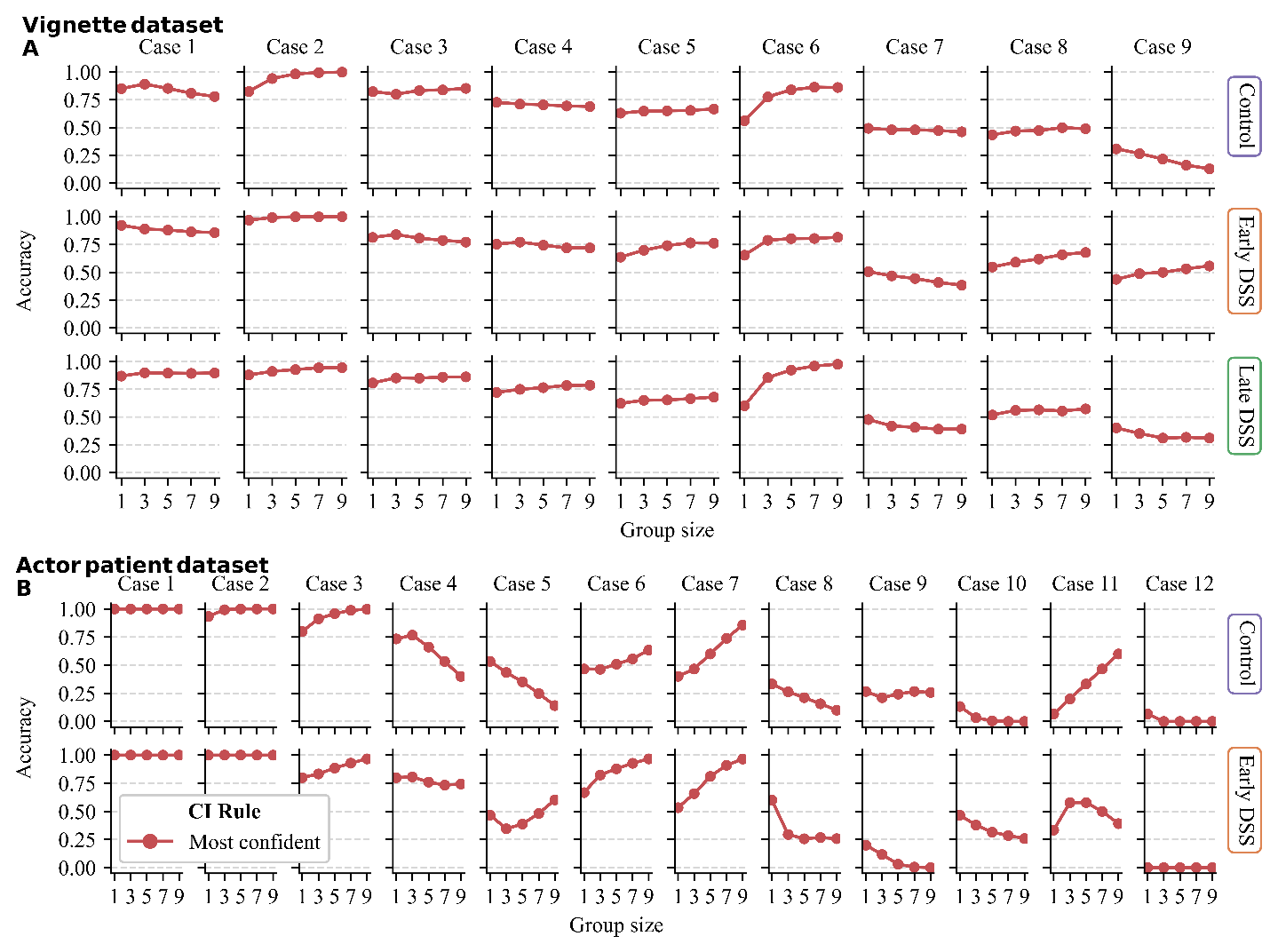


**Supplementary Figure 3**: Performance of the most-confident rule for each case and in each condition for both datasets. Within each dataset, cases are arranged (from left to right) based on the mean individual accuracy in the control condition, with the highest (lowest) mean individual accuracy on the left (right). This order is identical to Main Figure 3.


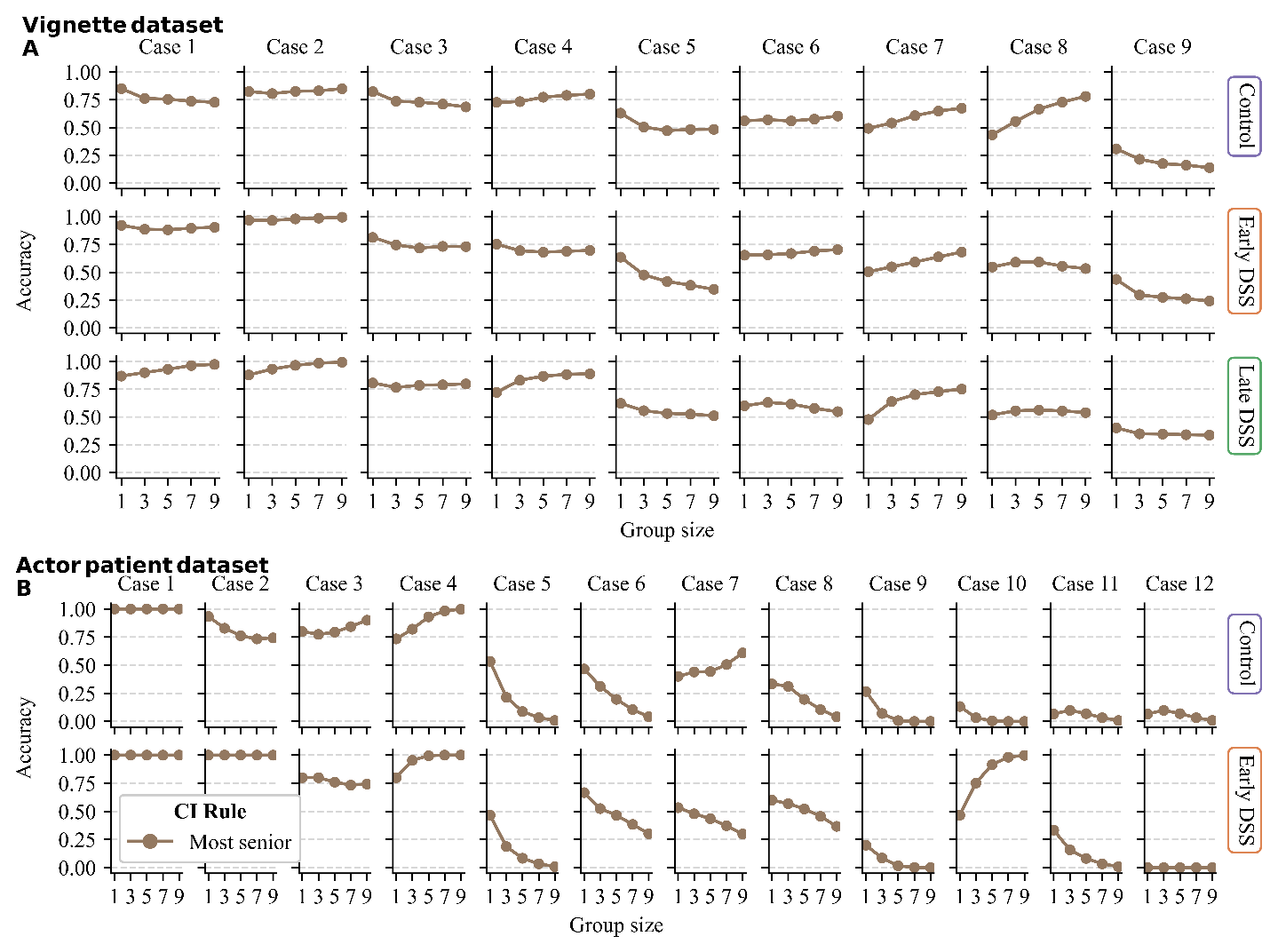


**Supplementary Figure 4**: Performance of the most-senior rule for each case and in each condition for both datasets. Within each dataset, cases are arranged (from left to right) based on the mean individual accuracy in the control condition, with the highest (lowest) mean individual accuracy on the left (right). This order is identical to Main Figure 3.

**Supplementary Table 1:** Results of the Bayesian mixed-level logistic regression model testing the effect of confidence, seniority and condition on accuracy in the vignette dataset. C = Condition.

|  | **Estimate** | **Est.Error** | **l-95% CI** | **u-95% CI** | **Rhat** | **Bulk_ESS** | **Tail_ESS** |
| --- | --- | --- | --- | --- | --- | --- | --- |
| **Intercept** | -0.54 | 0.66 | -1.88 | 0.78 | 1 | 4750 | 6054 |
| **Confidence** | 0.21 | 0.08 | 0.05 | 0.38 | 1 | 7425 | 6899 |
| **Seniority** | -0.01 | 0.01 | -0.04 | 0.01 | 1 | 8885 | 7093 |
| **C_Early** | -0.09 | 0.73 | -1.56 | 1.35 | 1 | 8350 | 7411 |
| **C_Late** | -0.82 | 0.7 | -2.18 | 0.53 | 1 | 7634 | 7009 |
| **Confidence:C_Early** | 0.11 | 0.12 | -0.12 | 0.33 | 1 | 8519 | 7212 |
| **Confidence:C_Late** | 0.12 | 0.11 | -0.09 | 0.34 | 1 | 7717 | 6949 |
| **Seniority:C_Early** | -0.02 | 0.02 | -0.05 | 0.02 | 1 | 9058 | 7401 |
| **Seniority:C_Late** | 0.03 | 0.02 | -0.01 | 0.07 | 1 | 9236 | 8029 |

**Supplementary Table 2:** Results of the Bayesian mixed-level logistic regression model testing the effect of confidence, seniority and condition on accuracy in the actor-patient dataset.

|  | **Estimate** | **Est.Error** | **l-95% CI** | **u-95% CI** | **Rhat** | **Bulk_ESS** | **Tail_ESS** |
| --- | --- | --- | --- | --- | --- | --- | --- |
| **Intercept** | 0.52 | 1.19 | -1.82 | 2.92 | 1 | 2670 | 4530 |
| **Confidence** | -0.01 | 0.11 | -0.24 | 0.21 | 1 | 6679 | 6587 |
| **Seniority** | -0.03 | 0.02 | -0.07 | 0.01 | 1 | 4941 | 6276 |
| **Condition** | -0.26 | 1.35 | -2.91 | 2.37 | 1 | 5412 | 6296 |
| **Confidence:Condition** | 0.07 | 0.17 | -0.26 | 0.4 | 1 | 5223 | 6085 |
| **Seniority:Condition** | 0.02 | 0.02 | -0.02 | 0.06 | 1 | 8251 | 6769 |
